# DMSO and Betaine Greatly Improve Amplification of GC-Rich Constructs in *De Novo* Synthesis

**DOI:** 10.1371/journal.pone.0011024

**Published:** 2010-06-11

**Authors:** Michael A. Jensen, Marilyn Fukushima, Ronald W. Davis

**Affiliations:** Stanford Genome Technology Center, Stanford University, Palo Alto, California, United States of America; Virginia Commonwealth University, United States of America

## Abstract

In Synthetic Biology, *de novo* synthesis of GC-rich constructs poses a major challenge because of secondary structure formation and mispriming. While there are many web-based tools for codon optimizing difficult regions, no method currently exists that allows for potentially phenotypically important sequence conservation. Therefore, to overcome these limitations in researching GC-rich genes and their non-coding elements, we explored the use of DMSO and betaine in two conventional methods of assembly and amplification. For this study, we compared the polymerase (PCA) and ligase-based (LCR) methods for construction of two GC-rich gene fragments implicated in tumorigenesis, IGF2R and BRAF. Though we found no benefit in employing either DMSO or betaine during the assembly steps, both additives greatly improved target product specificity and yield during PCR amplification. Of the methods tested, LCR assembly proved far superior to PCA, generating a much more stable template to amplify from. We further report that DMSO and betaine are highly compatible with all other reaction components of gene synthesis and do not require any additional protocol modifications. Furthermore, we believe either additive will allow for the production of a wide variety of GC-rich gene constructs without the need for expensive and time-consuming sample extraction and purification prior to downstream application.

## Introduction

Since the *de novo* synthesis of the suppressor transfer RNA gene was first reported three decades ago [1], our ability to engineer and assemble synthetic gene constructs has revolutionized the field of biomedicine [2]–[5]. Yet, despite our many achievements from assembling multi-kilobase plasmids to whole genomes [6], [7], *de novo* synthesis of GC-rich fragments remains a major obstacle namely because of secondary structure formation. Sequences populated with G repeats produce complex inter and intrastrand folding due to increased hydrogen bonding with neighboring guanines at their N-7 ring positions [8]. In PCR, this phenomenon is marked by the appearance of shorter bands following gel electrophoresis. These truncated versions of the target amplicon are primarily the consequence of arrest sites (hairpins) introduced into the template causing premature termination to polymerase extension [9]. In addition, mispriming and mis-annealing between template and compliment strands due to high melting temperature (*T*_m_) overlaps may contribute to incorrectly amplified gene constructs [10]. Because of these complications, GC-rich sequences are typically optimized by the researcher using web-based tools [11]–[14] that disrupt G repeats by choosing synonymous codons with lower *T*_m_s. However, there may be instances where nucleotide conservation is essential [15]–[18] particularly for non-coding regions where secondary structure functions to activate or repress transcriptional initiation [19]. While techniques are available to manage these difficult regions during PCR amplification of plasmid and genomic DNA [20], [21], to our knowledge no method for *de novo* synthesis of GC-rich templates has been clearly defined. The closest application we found was GeneDesign [22], which has the option to circumvent base rearrangement by adjusting the overlap between complimentary strands. While this can aid in ‘normalizing’ the overall *T*_m_ of less GC-rich sequences, synthesis of longer oligodeoxynucleotides (ODN)s is often required, and may necessitate costly purification.

As a cheap and effective approach to disrupting secondary structure formation and minimizing high *T*_m_ ODN overlaps in *de novo* synthesis, we explored the use of the more popular and often referenced chemical agents, Dimethyl Sulfoxide (DMSO) [23], [24] and betaine [25], [26] during both the assembly and PCR amplification steps in conventional gene synthesis. These isostabilizing agents facilitate strand separation of double helix DNA by altering its melting characteristics. For example, betaine, an amino acid analog with both positive and negative charges close to neutral pH, acts to equilibrate the differential *T*_m_ between AT and GC base pairings; DMSO on the other hand, acts by disrupting inter and intrastrand re-annealing.

In this study, we compared the effects of these additives in the construction of two GC-rich gene fragments implicated in tumorigenesis, the Insulin-like Growth Factor 2 Receptor (IGF2R) [27], [28] and V-raf murine sarcoma viral oncogene homolog B1(BRAF) [29]–[31]. DMSO and betaine were also chosen because of their previously reported success in PCR amplification of the IGF2R gene fragment from a vector [25]. However, for our purposes, IGF2R and BRAF were chemically synthesized and assembled *in vitro* by pooling overlapping, single-stranded ODNs using two conventional methods, the Polymerase Chain Assembly (PCA) [32] and the Ligase Chain Reaction (LCR) [33]. For a typical PCA reaction, assembly is done with one or two pre-PCR steps where single-stranded ODNs prime off each other, building up to the full-length product; 40 bp ODNs are designed (no gaps) with 20 bp overlap between template and compliment strands where a 3′ recess allows for polymerase binding and strand propagation. ODNs for LCR are the same as those for PCA except that each strand is 5′ phosphorylated for ligation. In this case, complimentary ODNs are denatured and annealed over several cycles for optimum strand alignment. A final round of PCR is then employed in both methods to amplify the target product using outside primers.

Here we report that DMSO and betaine greatly improve *de novo* synthesis of IGF2R and BRAF gene fragments generated from both PCA and LCR methods of assembly. Though we only tested two genes, incorporation of either additive could aid in the construction of most GC-rich sequences. Protocol manipulation of standard conditions is also unnecessary due to the isostabilizing properties of these additives. Even without the need for nucleotide conservation, this application saves a great deal of end-user time not having to re-design and codon optimize ODNs prior to synthesis. As such, the possibility of manually introducing sequence error is also limited; one mismatch, deletion or insertion could lead to a frame-shift or other gene lethality. Furthermore, DMSO and betaine are very inexpensive, easily obtainable and highly compatible with other biological agents, which make them ideal for any gene synthesis assay.

## Materials and Methods

### IGF2R and BRAF gene fragment designs

Sequences (5′–>3′) for IGF2R (bases 32–548) and BRAF (bases 1–512) were taken from the National Center for Biotechnology Information database (ACCESSION: NM_000876 and NM_004333, respectively). They were then entered into Gene2Oligo (http://berry.engin.umich.edu/gene2oligo/index.html), which cut both constructs up into 40 bp fragments with 20 bp hybridizable overlap between the +/− strands [14]. Though this program has the option of calculating the optimum length of overlap given a target uniform *T*_m_, no such parameters were defined for either construct. ODN *T_m_* values were calculated with Gene2Oligo using the Nearest Neighbor model.

### Synthesis of IGF2R and BRAF constructs

ODN synthesis of both genes was done in-house (Stanford Genome Technology Center) with a 3900 DNA synthesizer (Applied Biosystems) using 1000 Å CPG columns (Biosearch Technologies) for a 50 nmole-scale synthesis. Cycle conditions were similar to the manufacture's recommended protocol, which included the following reagents: deblock (3% TCA/DCM) (AiC), acetonitrile, 0.02 M oxidizing solution, cap A/B, 0.1 M solutions of dA, dC, dG and dT (Proligo), and 0.25 M 5-Benzylthio-1H-tetrazole (Glen Research). Post-synthesis steps included ODN cleavage from the support followed by base-deprotection overnight at 55°C with ammonium hydroxide (28–30%) (J.T. Baker). After lyophilization, ODNs were resuspended and the optical density for each was measured at 260 nm using a Spectramax 384 Plus 96-well plate reader. All ODNs were then normalized to 100 µM in water and analyzed for purity using reverse-phase HPLC (Transgenomic WAVE system).

### Assembly and PCR amplification

For PCA, unmodified +/− strands were pooled together (100 µM), where 1 µl was added to High Fidelity (HF) Advantage polymerase mix (Clontech) according to the manufacture's recommended protocol (DMSO and betaine were not included in this kit); samples were then run on a Veriti 96-well thermal cycler (Applied Biosystems) through two iterations using the following parameters: 94°C/5 min | 20* [94°C/15 sec | 55°C/30 sec | 68°C/60 sec], where 1 µl from the first reaction was transferred to the second step (PCR components were replenished to a 20 µl final volume). Five µl from the last step were then taken for PCR amplification.

For LCR, ODNs were pooled separately into +/− strands (100 µM). Each set was enzymatically 5′ phosphorylated by adding 3 µl DNA to 41 µl water, 5 µl 10X T4 DNA ligase buffer with ATP and 10 U T4 Polynucleotide Kinase (NEB). Samples were incubated at 37°C for 30 min then heat-inactivated at 60°C for 20 min. Twenty-five µl each of +/− strands were desalted using Micro Bio-Spin 6 Chromatography columns (Bio-Rad), then pooled together. Two µl (∼12 pmoles) of the phosphorylated product were added to 41 µl water, 5 µl Ampligase 10X Reaction Buffer and 2 µl (10 U) of Ampligase (Epicentre). The ligation reactions were cycled at 21* [95°C/1 min |∧ 70°C/4 min] then cooled to 4°C (∧ −1° per cycle). Following assembly with both PCA and LCR, target product was then selected for through PCR amplification using the forward and reverse primers (5′−>3′) TCCCGCTCCGTCTCCACCTCCGC | ACAGGAAGGCAATGCTGCTCTGGA (IGF2R) [25] and CGCCTCCCTTCCCCCTCCCC | ACTTGGGGTTGCTCCGTGCC (BRAF). PCR conditions using HF Advantage were as follows: 94°C/5 min | 25* [94°C/15 sec | 55°C/30 sec | 68°C/60 sec] 68°C/5 min. For gel analysis of final product, 2 µl 6x Orange Loading Dye (Fermentas) were added to 10 µl of each PCR sample. Five µl O' GeneRuler 1 kb DNA Ladder Plus (Fermentas) were used in all cases for band size comparison. Samples were electrophoresed through a 1.25% SeaKem LE Agarose (Lonza) gel at 80 V for 45 min, stained with ethidium bromide, then visualized using a standard UV imager at 302 nm.

### Additives

DMSO (99.9%) and betaine (5 M) (Sigma-Aldrich) were added in varying concentrations to select PCA, LCR and PCR steps. Where required, water was replaced with the necessary amount of either additive to generate 1–10% DMSO or 0.5–2.5 M betaine per 20 µl reaction (or 50 µl for LCR assembly).

## Results

IGF2R was chosen as a template to determine if the same additives, DMSO and betaine used to successfully amplify the fully formed gene fragment [25] could also be employed to aid in building and amplifying it from a pool of overlapping, single-stranded ODNs. The BRAF gene fragment was also synthesized *de novo* for quality comparison given the same conditions as IGF2R. Particular attention was paid to the 5′ region of IGF2R (81.5% GC-rich between bases 1 and 260), which includes the non-coding element [34]. The rest of the gene fragment from bases 261 to 517 averages 44.4% GC. With respect to the hybridization map generated from Gene2Oligo [14], *T*_m_s for the 20 bp overlaps average 87.0°C (first 260 bases) with a maximum of 92.6°C. For the BRAF gene, the first 183 bases are the most GC abundant at 78.1% (83.7°C average *T*_m_) and 43.2% for bases 184–512.

**Figure 1:**
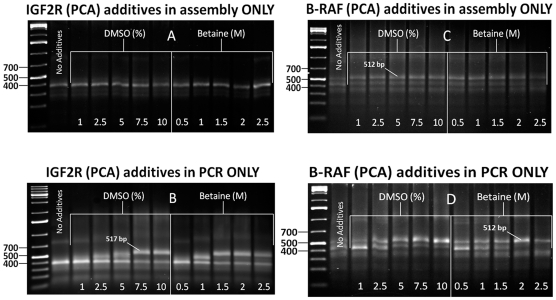
Agarose gel images showing the effects of DMSO and betaine during the PCA assembly (A and C) and amplification (B and D) of IGF2R and BRAF gene fragments. Based on a 20 µl reaction volume, both additives were introduced with increasing percentage (%) for DMSO, and molarity (M) for betaine; ‘No Additive’ lanes correspond to the control samples. A 1 kb DNA ladder in the outermost lanes marked at 400, 500 and 700 bp indicates the area of highest product band population. IGF2R and BRAF full-length gene fragments are shown at 517 bp and 512 bp, respectively.

To determine at what concentration either DMSO or betaine improved full-length product generation of IGF2R and BRAF in *de novo* synthesis, we ran 1-10% DMSO and 0.5–2.5 M betaine gradients separately on both gene fragments. First, we tested if these additives had any effect on the assembly stage alone using the PCA method (Fig. 1A and C). Here, samples were ethanol precipitated to eliminate carry-over from assembly to the amplification step. Figure 1A shows no observable effect on target formation of the IGF2R gene fragment at 517 bp, whereas the slightly less GC-rich BRAF (Fig. 1C) has minor product formation at 512 bp at approximately the same intensity spanning the entire gradient; results for both genes are comparable to the control samples where DMSO and betaine were not added. IGF2R and BRAF showed the most improvement in target-specific amplification when processed with additives in the PCR step alone (Fig. 1B and D). Overall, there is a marked disappearance of truncated species with a simultaneous formation of target product as the concentration of each additive is increased (maximum effect for both genes at about 10% DMSO or 2 M betaine).

**Figure 2:**
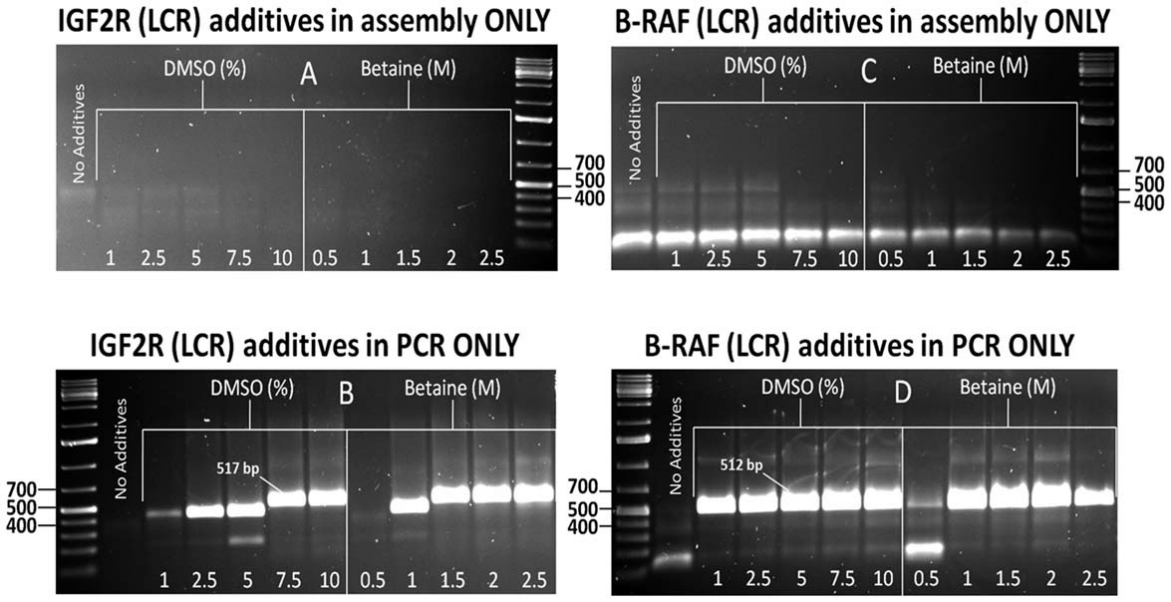
Agarose gel images showing the effects of DMSO and betaine during LCR assembly (A and C) and amplification (B and D) of IGF2R and BRAF gene fragments. Based on a 20 µl reaction volume, both additives were introduced with increasing percentage (%) for DMSO, and molarity (M) for betaine; ‘No Additive’ lanes correspond to the control samples. A 1 kb DNA ladder in the outermost lanes marked at 400, 500 and 700 bp indicates the area of highest product band population. IGF2R and BRAF full-length gene fragments are shown at 517 bp and 512 bp, respectively.

Next, we assembled IGF2R and BRAF gene fragments using LCR to compare product formation with that generated from the PCA method; here, DMSO and betaine were applied in the same concentration gradients (Fig. 2A–D). It was rationalized that tiling ODNs through ligation would better stabilize the template strands prior to their amplification. Similar to PCA, we introduced additives only during the ligation assembly step prior to ethanol precipitation. Neither DMSO nor betaine showed any marked influence on product formation using the stock polymerase mix for PCR when compared with the control samples (Fig. 2A and C). Only when additives were introduced during the amplification step did target yield and specificity greatly improve (Fig. 2B and D).

For both gene fragments amplified from either PCA or LCR, locations of polymerase arrest correlate with the GC-rich regions of each construct. With PCA of IGF2R, for example, we find strong band formation just below 400 bp (Fig. 1A and B) as would be expected since this region overlaps with the gene's non-coding element from bases 1–147 [34]. Similarly, the GC-rich region of BRAF, which is within the first 183 bases, coincides with the presence of truncated species starting at about 329 bp (Fig. 1C and D). On the other hand, in both cases these prematurely terminated PCR bands are negligible in samples processed using the ligase-based method where additives were introduced into the amplification step alone (Fig. 2B and D).

## Discussion

We have shown that DMSO and betaine greatly improve *de novo* synthesis of two GC-rich gene fragments, IGF2R and BRAF without having to modify nucleotide composition. This is particularly important when studying non-coding elements in cancer gene research where base conservation may be critical to structural function in malignant cell types. And because no optimum conditions for *de novo* synthesis of GC-rich genes have been previously reported, we compared both conventional methods of assembly, PCA and LCR in the presence and absence of DMSO and betaine during the construction of IGF2R and BRAF. We discovered that either chemical agent introduced into the assembly steps alone (PCA and LCR), despite concentration, had no effect on target generation when amplified with the stock polymerase mix; only when DMSO and betaine were added to the amplification step did they show a marked improvement, especially in the case of LCR.

For the LCR samples (Fig. 2B and D), increased target yield and specificity of both IGF2R and BRAF genes compared with PCA (Fig. 1B and D), is due to the higher stringency of the ligation method; only those overlapping fragments that are perfectly matched are ligated together [35]. Thus, protruding hairpin structures cause misalignment of template and compliment strands, which subsequently do not tether to form a stable duplex. Proof-reading Exonuclease I activity in commercial polymerase mixes (such as HF Advantage) further degrades all unpaired, single-stranded ODNs, thereby “cleaning up” the reactions [36]. In contrast, multiple bands generated in PCA (Fig. 1B and D) are also the consequence of non-specific priming of overlapping ODNs during assembly, and are unavoidable without cycle optimization [37]. For these reasons, the added stability of LCR product accounts for the increased target band specificity and high yield amplification without the need for either DMSO or betaine during assembly.

Furthermore, our results for *de novo* synthesis of IGF2R are comparable to those obtained by Frackman *et al*. who PCR amplified IGF2R from a vector using the same additives [25]. It is the assembly from a pool of single-stranded, GC-rich ODNs that makes construction of genes with non-coding elements so challenging. There are any number of ways neighboring guanines can interact to form secondary structure such as when a single strand folds onto itself (unimolecular), two separate strands interact (bimolecular), or where four different molecules join to form a quadrimolecular G-complex [38]. However, with introduction of either DMSO or betaine during the amplification process, much of the secondary structure caused by G-G interaction is disrupted. It is because of their isostabilizing properties that they are able to minimize if not eliminate problems of low yield and aberrant band formation associated with polymerase arrest and sequence mis-annealing. This in turn provides the end-user with a larger amount of working construct to maximize the number of reactions per experiment; and though target product was formed with both processes, LCR gave the highest yield with little to no background. Therefore, with the ligase-based method one avoids having to gel extract and purify samples prior to their use in downstream application.

While for this study we chose IGF2R and BRAF as representative genes with moderately high GC-rich content, there may be sequences of interest that contain more consecutive guanines per stretch (>90% GC). In this case, other chemical and biological additives are available that might prove more effective than either DMSO or betaine. These include formamide, glycerol, NP-40, Tween 20, trehalose, EcoSSB and 7-deaza-2′-deoxyguanosine 5′ triphosphate (dc^7^GTP) [23], [39]–[43]; dc^7^GTP works differently than the other additives in that hydrogen bonding between neighboring guanines is minimized due to nitrogen displacement from position 7 of the base ring to position 8. It has also been reported that *Thermococcus litoralis* exo- (*Vent* exo-) DNA polymerase has helped resolve GC-rich sequences better than the *Thermus aquaticus* (*Taq*) DNA polymerase variety used in this study [44]. Because DMSO and betaine showed substantial improvement in amplification of the IGF2R and BRAF gene fragments, we limited our testing to these particular chemical agents. If on the other hand, either had proven inefficient, further exploration into any one of the aforementioned chemical agents/additives would have been warranted. Moreover, the PCA, LCR and PCR methods applied in this work have not been modified in any way to account for increased structure *T*_m_. We wanted to see how each process would be affected by the addition of either DMSO or betaine alone. It is likely our results might have varied if we had altered any of the cycle parameters, especially those for PCA; increasing the annealing temperatures, for example, could have improved target yield and specificity by generating less spurious PCR product.

### Conclusion

Secondary structure formation and mispriming in *de novo* synthesis of GC-rich constructs greatly inhibits our flexibility to investigate important genes and their non-coding elements, especially in cancer research. Because no application currently exists that defines a method for maintaining nucleotide composition for structural function in gene synthesis, we explored the use of cheap and readily available chemical additives, DMSO and betaine to aid in the production of GC-rich constructs. For this study, we chose two GC-rich gene fragments implicated in tumorigenesis, IGF2R and BRAF to determine the effectiveness of either DMSO or betaine in disrupting secondary structure formation and minimizing high *T*_m_ ODN overlaps. While these additives had no benefit during the assembly steps of either process (PCA and LCR), we have shown they substantially improve gene target-specific amplification and yield when added to the PCR step alone. And because of their compatibility with routine gene synthesis constituents, DMSO and betaine may be well suited in aiding *de novo* synthesis of a wide range of GC-rich gene constructs and their non-coding regions. Furthermore, we demonstrated that LCR is the preferred method of assembly, yielding the highest amount of target product with the cleanest background. This in turn gives the end-user plenty of working material without the need for expensive and time-consuming sample extraction and purification between applications.
